# Acupuncture's Effects in Treating the Sequelae of Acute and Chronic Spinal Cord Injuries: A Review of Allopathic and Traditional Chinese Medicine Literature

**DOI:** 10.1093/ecam/nep010

**Published:** 2010-10-25

**Authors:** Peter T. Dorsher, Peter M. McIntosh

**Affiliations:** Department of Physical Medicine and Rehabilitation, Mayo Clinic, 4500 San Pablo Road, Jacksonville, Florida 32224, USA

## Abstract

Each year, there are an estimated 12 000 individuals who sustain a spinal cord injury (SCI) in the United States. Improved understanding of the pathophysiology of SCI and its sequelae has over the past 50 years led to the development of medical treatments (especially urologic) that have enhanced short- and long-term survival from these injuries. The prevalence of individuals with SCI in this country is ~250 000 individuals; and beyond the incalculable personal consequences of these devastating neurologic injuries, substantial direct and indirect societal costs result from the sequelae of SCI including paralysis, sensory loss, chronic pain, decubiti and bladder and/or bowel incontinence. The purpose of this treatise is to review the allopathic and traditional Chinese medicine (TCM) literature available through MEDLINE, PubMed and eCAM search engines that discuss the potential uses of acupuncture to treat acute and chronic spinal cord injuries and their sequelae, and present the neurophysiologic mechanisms for acupuncture's beneficial effects. There is evidence that use of electroacupuncture in acute SCI may significantly improve long-term neurologic recovery from these injuries both in terms of motor, sensory and bowel/bladder function with essentially no risk. Acupuncture may even improve neurourologic function in individuals with chronic SCI, and help with management with chronic pain associated with these injuries.

## 1. Introduction

Due to advances in critical care medicine and spine surgery techniques in the last few decades, more individuals survive severe trauma that result in spinal injuries. The most devastating sequelae of spinal trauma are spinal cord injuries (SCI). The annual incidence of SCI in the United States is *∼*12000/year, with a prevalence of over 250000 persons [1]. Nearly 80% of individuals sustaining SCI are male; and since 2000, their average age at injury has increased to an average of 39.5 years with 11.5% of SCI individuals being over the age of 60 [1]. Improved urologic care in the last 50 years has helped prevent the progressive renal failure that previously resulted from recurrent urinary tract infections and hydronephrosis related to bladder dyssynergia and high intravesicular pressures, so that life expectancy of individuals with paraplegia is only about 6 years shorter than their non-SCI peers and only about 10 years shorter for those with low (C5–C8) quadriplegia [1].

Beyond the incalculable personal consequences of SCI, the medical care costs of individuals sustaining SCI are substantial. The lifetime medical costs of a 25-year-old person with high (C1–C4) quadriplegia is estimated to be over $3 million, over $1.7 million for those with low quadriplegia, over $1 million for paraplegics and over $680000 for individuals sustaining incomplete SCI [1]. These figures do not include nearly $62000/year indirect losses with respect to wages, fringe benefits and productivity [1].

Interventions that improve neurologic recovery from acute SCI would not only potentially improve the quality and quantity of their lives, but would also reduce their health care needs and thus costs. Methyprednisolone use acutely after SCI was the first intervention that appeared to have some influence on the neurologic recovery from SCI [2]. Though statistically significant improvements in motor scores were reported in those receiving methylprednisolone, the clinical significance of these improvements was questionable, and no improvements in myotomal levels were noted [2]. GM-1 ganglioside [3], 4-aminopyridine [4], nimodipine [5] and naloxone [6] trials likewise failed to improve neurologic outcomes when administered for acute SCI.

The purpose of this study is to review the allopathic and traditional Chinese medicine (TCM) literature examining the use of acupuncture for treating SCIs and their sequelae. Due to significant methodological differences, the TCM reports on acupuncture for treating SCIs will be covered separately. The eCAM, MEDLINE and PUBMED databases were searched with the terms “acupuncture" and “SCI". Reports in English or with an English abstract relevant to the use of acupuncture to treat the sequelae of acute and chronic SCI were reviewed for this study.

## 2. Motor and Sensory Recovery

Less than 1% of persons with acute SCI experience complete neurologic recovery by hospital discharge [1]. Wong et al. [7] examined the effect of electroacupuncture on neurologic recovery in 100 subjects with acute ASIA A/B traumatic SCIs. Fifty subjects received only standard rehabilitation care, while the other 50 subjects received standard care plus electroacupuncture. Those subjects received 75 Hz stimulation of surface electrodes at acupuncture points SI-3 (lateral fifth metacarpal) and BL-62 (inferior to lateral malleolus) with auricular acupuncture five times weekly for 30 min from the time of SCI/surgery to rehabilitation discharge [7]. The cohort receiving electroacupuncture had statistically and clinically significant improvements in their neurologic outcomes at the 1 year mark, compared to those who received standard rehabilitation care [7]. ASIA motor scores were significantly improved in the electroacupuncture group compared to the control group (74.2 versus 52.3, mean) as were pinprick sensory scores (90.0 versus 69.8, mean), light touch sensory scores (92.5 versus 70.5, mean), and total FIM scores (106.9 versus 88.7, mean). Of the 50 subjects in the control group receiving only standard rehabilitation care, 32 (64%) remained ASIA A or B 1 year post injury follow up, 8 (16%) improved to ASIA C, six (12%) improved to ASIA D and four (8%) improved to ASIA E [7]. In contrast, the cohort who received electroacupuncture along with standard rehabilitation care showed much better neurologic outcomes. Only 11 (22%) of the electroacupuncture group remained ASIA A or B 1 year post injury, while 10 (20%) improved to ASIA C, 8 (16%) improved to ASIA D and 21 (42%) improved to ASIA E [7]. Seven subjects with ASIA A injuries receiving electroacupuncture improved to ASIA C/D/E at the 1 year mark, compared to none in the control group [7]. Of 22 subjects, 16 with ASIA B injuries receiving electroacupuncture improved to ASIA E at the 1 year follow up, and the other six to ASIA D, while in the control group only 4 of 18 improved to ASIA E status at the 1 year mark and six to ASIA D [7].

Thus, study of Wong et al. suggests a much larger effect of electroacupuncture on ultimate neurologic recovery from acute SCI than any pharmacologic intervention to date. The acupuncture points chosen are described to activate the Governor Vessel meridian, which is described to ascend through the sacrum and spinal column to the base of the skull [8, 9]. The ankle acupuncture point BL-62 has sensory innervation by the sural nerve, a branch of the sciatic nerve, while the hand acupuncture point SI-3 is innervated by the ulnar nerve. Electrical stimulation at this relatively high frequency (75 Hz) is known to release enkephalins and dynorphins at the spinal cord level [10], providing a putative endogenous mechanism for limiting the damage to spinal cord neurons and axons following trauma. This landmark study should be replicated by another center(s), and if these impressive results are confirmed, then this treatment should be integrated into the acute management of SCIs.

The study of Wong et al. [7] does not score highly on the Jadad scale, as it does not describe the randomization process for subjects entered in the study, and it is not double-blinded (Table 1). The actual study data, however, shows that the acupuncture and control groups were well matched at study entry in terms of patient ages, duration of SCIs, male/female distributions, quadriplegia/paraplegia proportions, ASIA classifications and FIM scores. The same nurse practitioner did all patient examinations, though the study does not report whether or not the nurse practitioner was blinded to the type of intervention of the subject patients. Double blinding was not an element of the study, as it compared usual rehabilitation care to usual rehabilitation care plus a standardized electroacupuncture intervention.

Electroacupuncture in an animal model of incomplete acute SCI [20] led to more rapid neurologic recovery of proprioception (>2 times more rapidly) and motor function (>3 times more rapidly), though addition of corticosteroid to the electroacupuncture further enhanced the rate of neurologic recovery.

## 3. Bladder Dysfunction

Urinary incontinence related to SCIs has been reported to have a poor long-term prognosis for recovery [21–23]. Prior studies document that up to 41% of patients with normal neurologic examinations after thoracolumbar spinal injuries demonstrate neurogenic bladders on urodynamic testing [24]. Bulbocavernosus reflex and saddle sensation testing are specific but not sufficiently sensitive to clinically select those patients with neurogenic bladder [24]. A retrospective study by Weiss et al. [25] found that SCI patients without perianal pinprick sensation at initial evaluation never regained volitional voiding, and lack of toe proprioception at initial evaluation strongly predicted failure to recover volitional voiding. A recent case study, however, documented a subject with traumatic SCI who had urinary incontinence with absent saddle sensation at initial presentation who became completely continent of urine and able to void volitionally after treatment with electroacupuncture [26].

Traditionally, treatment of UMN neurogenic bladder incontinence has included behavioral interventions (fluid schedules and regular voiding attempts), bladder catheterization and a variety of medications including anti-cholinergic and alpha blocker agents [23].

There have been a limited number of research studies examining the use of acupuncture to treat neurogenic bladder dysfunction due to UMN SCI. For patients with acute SCIs (<1 month), Cheng et al. [13] demonstrated that 20 Hz electroacupuncture at CV-3, CV-4 and BL-32 helped acute SCI patients with neurogenic bladder achieve balanced (continent, catheter free) bladder function 33% more rapidly than those receiving only usual care including medications and training in self catheterization. CV-3 and CV-4 are on the midline in the lower abdomen over the location of the bladder and influence parasympathetic nervous system, while BL-32 is at the second sacral foramen. Recall that the sacral plexus at S2–S4 provides parasympathetic innervation to the bladder as well as innervation of the external urinary sphincter *via* the pudendal nerve. The acupuncture intervention in study of Cheng et al. [13] did not improve bladder function in subjects having complete upper or lower motor neuron SCIs, and it was most efficacious if initiated within 3 weeks of the spinal injuries. That study examined only 60 (out of 80, total randomized) subjects who ultimately achieved balanced bladder status, and its analysis examined both upper motor and lower motor neuron neurogenic bladders [13]. This study had a Jadad score of 2, as it did not discuss the method of randomization of subjects and was not double blinded, comparing electroacupuncture plus usual bladder retraining to usual bladder training alone (Table 1). Wong et al. [7] demonstrated that electroacupuncture at SI-3 and BL-62 in conjunction with auricular acupoints produced enhanced recovery of bladder function in patients with acute SCI. At 1 year post SCI, subjects receiving the acupuncture intervention demonstrated statistically significant improvements in their bladder functional independence measure (FIM) scores to a mean score of 3.02 compared to subjects in the control group whose mean bladder FIM score was 1.49 [7]. An FIM bladder control score of 3 indicates a subject has no incontinence episodes, and is independent with use of a catheter or uses medication for control, while a score of 1.5 would indicates that the subject has urinary incontinence episodes nearly every day and requires significant physical assistance with bladder management.

Even in subjects with chronic SCI, acupuncture may have a beneficial effect in treating neurogenic bladder dysfunction. Honjo et al. [14] performed manual stimulation of point BL-33 (third sacral foramen) weekly for a month in 13 subjects with chronic SCI, which resulted in resolution of urinary incontinence in 15% of subjects and at least 50% improvement in incontinence symptoms in another 46% [14]. The average bladder volume increased *∼*2-fold after four weekly treatments, and was still 70% improved one month following the last acupuncture treatment [14]. Recall that the parasympathetic outflow to the bladder occurs from the sacral plexus at the S2–S4 spinal levels as does the innervation to the external urethral sphincter via the pudendal nerve. This study had a Jadad score of 1 as it was not randomized and the intervention was not blinded (Table 1).

## 4. Neurogenic Bowel

Incontinence of bowel following SCI can have devastating consequences on re-integration of SCI individuals into work and social situations. The natural history of recovery of bowel function following SCI is not favorable [27]. Traditional treatments of neurogenic bowel dysfunction have included use of stool softeners and fiber to achieve proper stool consistency, then regular use of suppositories or enemas to promote adequate evacuation of the lower colon to minimize risk of gross incontinence or stool leakage.

Wong et al. [7] provide the only study in the literature that provides objective evidence of an intervention which improves the natural history of neurogenic bowel dysfunction following acute SCI, by demonstrating electroacupuncture produced statistically significant (*P* = .001) improvement in bowel FIM scores 1 year post injury compared to control group receiving no acupuncture (mean score 3.42 versus 1.70, resp.). A bowel management FIM score of 3 means no incontinence or assistance from another person for bowel care, while a score of 1.7 indicates the need for assistance with bowel care and some episodes of fecal incontinence, though not on a daily basis. Thus, this represents a clinically significant improvement in bowel incontinence.

## 5. Pain

Over 80% of individuals sustaining SCI experience pain [28]. Approximately 40% of individuals sustaining traumatic or non-traumatic SCI experience neuropathic pain that in two-thirds of cases adversely affects their daily lives, and over 60% of these individuals report experiencing the pain below the level of the spinal injury [29]. Musculoskeletal pain results from repetitive use of the upper extremities for transfers and wheelchair mobility [30, 31].

Nayak et al. [16] studied 22 patients with SCI who suffered moderate to severe pain of at least six months' duration and received a course of 15 acupuncture treatments over a seven and a half week period after an equivalent assessment period without treatment. Ten patients (46%) showed improvement in pain intensity and pain sequelae after treatment, whilst six patients (27%) reported an increase in pain, that was still present 3 months after treatment. The researchers conclude that acupuncture may provide pain relief for at least a subgroup of individuals with SCI and that future research is needed to determine what part of this effect is due to acupuncture [16]. This study had a Jadad score of 1 as it was not randomized and the study intervention was not blinded (Table 1).

Rapson et al. [17], in a retrospective analysis, reported two-thirds of 36 SCI patients with central pain treated with electroacupuncture improved. The type and level of SCI did not predict improvement with acupuncture nor did duration of pain symptoms [17]; but subjects with bilateral, burning pain below the SCI lesion were the most likely to improve (*P* = .005). This study had a Jadad score of 0 as it was not randomized, the study intervention was not blinded, and was a retrospective clinical series with no discussion of dropouts (Table 1).

Dyson-Hudson et al. [18] studied the effects of acupuncture in 17 wheelchair-using SCI subjects having chronic musculoskeletal shoulder pain. Ten treatments of acupuncture or minimal needling of non-acupuncture points were performed [18], and both groups had statistically significant reductions in shoulder pain decreased significantly with treatment (66% and 43% reduction, resp.). There was no statistically significant difference between the two groups (though study power was low), but there was a medium effect size associated with the acupuncture treatment [18]. This study had a Jadad score of 5, reflecting the investigators' adherence to CONSORT guidelines for reporting clinical trials (Table 1).

Acupuncture is known to release endogenous opioids including endorphins, and its pain relieving effects can be blocked by naloxone [32, 33]. Overall, acupuncture may be a useful adjunct to treat neuropathic and/or musculoskeletal pain following SCI.

## 6. Autonomic Hyper-Reflexia

Though no studies have looked at the use of acupuncture to treat autonomic dysreflexia associated with SCI that can occur with cord lesions at or above T8, Averill et al. [19] studied the effects of acupuncture needle insertion above and below the spinal injury level of 15 patients with SCI who were at risk for autonomic dysreflexia. Though blood pressures overall remained stable over 15 treatment sessions, three individuals did have acute systolic blood pressure elevations (>20 mmHg or higher) that suggested incipient dysreflexia. The authors concluded that blood pressure should be monitored in at risk SCI patients receiving acupuncture [19]. This study had a Jadad score of 1 as it was not randomized and the study intervention was not blinded (Table 1).

A recent study by Flachskampf et al. [34] provided the first rigorous, randomized trial of the use of acupuncture to treat hypertension. This study's results suggest that acupuncture produces blood-pressure reductions comparable to those produced by ACE-inhibitor monotherapy or aggressive lifestyle changes, including radical salt restrictions, in mild to moderate hypertension. Acupuncture's beneficial effects dissipated after 3 months, however, indicating the need for ongoing treatments to maintain blood-pressure reductions [34]. The degree of blood-pressure reductions from this intervention were too small to be of clinical utility in autonomic dysreflexia, but other acupuncture treatment protocols such as needling the sympathetic switches [35] should be studied to determine if acupuncture could help treat the blood-pressure elevation associated with autonomic hyper-reflexia non-pharmacologically.

## 7. Decubiti

There are no experimental acupuncture studies that specifically examine its effect on decubiti, though Wang [11] reported that direct current stimulation with 3 V battery across a saline soaked gauze pad has “long been used … to treat paraplegic bed sores". Electrical stimulation around the site of decubiti has been demonstrated to enhance the rate of wound healing [36, 37]. Thus, electroacupuncture around a decubiti would be expected to enhance the rate of decubitus wound healing, and should be the subject of a future study.

## 8. Sexual Dysfunction

There are no acupuncture studies specifically examining its effect on sexual functioning after SCI. Wang [11] reported that sexual dysfunction related to SCI can be “cured" with electroacupuncture in 92.5% of those individuals, though no supporting evidence was provided in that report.

## 9. Traditional Chinese Medicine Literature on Acupuncture for SCI

There are two main reports most frequently cited regarding the use of TCM to treat SCI and their sequelae. These are the retrospective analyses by Wang [11] in 1992 and Gao et al. [12] in 1996. These are reported here as a separate section since both these studies reflect the methodological differences present throughout the TCM literature. Literature in this time interval by TCM practitioners in Asia reflects acupuncture's accepted efficacy there; so clinical studies usually do not include sham treatment arms which would be considered unethical.

Wang [11] reported a survey of TCM treatment of traumatic paraplegia which the paper defined as “sensory disturbance in the lower limbs and the accompanying loss of motor function along with urinary and defecation dysfunction." The paper actually discusses acupuncture point selection for treating the sequelae of quadriplegia and paraplegia, as well as upper and lower motor neuron injuries. The patient cohort is not fully described, and also appears to include individuals with complete and incomplete SCI. Electroacupuncture performed 12 times per week for 3 months was stated to have 100% “effectiveness" in treating urinary and fecal disturbances due to SCI, but no description of what “effectiveness" means [11]. No urodynamic confirmation of neurogenic bladder dysfunction is provided before or after acupuncture intervention. This survey concluded that “electric needling of the spinal cord … is the treatment of choice … by its marked therapeutic effect on the recovery and re-establishment of motor, urinary bladder, and sexual functions, with a total cure rate of 92.5%" [11]. The discussion section of this report, however, also intimates that complete SCI does not respond to acupuncture intervention [11].

Gao et al. [12] reported their retrospective analysis of 261 persons with “complete traumatic paraplegia"; but similar to the report of Wang [11], the patient descriptions appear to include those with quadriplegia and paraplegia as well as upper and motor lower neuron weakness. It is not clear from this report's descriptions that the subjects had complete injuries as saddle sensation was not reported and their subjects had “loss of flexor muscle ability to extend" [12]. Fifty six of the subjects had duration of SCI <1 year, and 24 of those <6 months. Of the eight subjects who were “essentially cured" by acupuncture with the ability to “walk freely without any sort of help … almost voluntary urination" [12], their injuries were all <6 months duration and thus may instead reflect spontaneous neurologic improvement of their injuries. 35% of subjects were reported to have experienced “marked effectiveness" from treatment defined as “partial recovery of the functions of the nervous system" with ability to “walk on crutches" and “restoration of bladder reflex"; and 57% were “improved" defined as “some improvement of the functions of the nervous system, including movement and defecation and/or urination" [12]. The lack of adequate descriptions of the subjects and objective data/measurements of their function makes interpretation of these results impossible.

One study from the TCM literature with an English abstract available by Zhou et al. [15] examined the effect of electroacupuncture on urinary retention due to SCI. Out of 84 study subjects, 46 had received electroacupuncture, which produced a positive effect on their urinary retention in 82.6% (versus 63.2% in control subjects), and a urinary retention “cure" rate of 43.5% (versus 23.7% in controls). The abstract did not delineate the acuity of the SCI, their types/levels, or other details of the subjects studied or interventions provided.

Another TCM study with an English abstract by Yu [38] reported that transcutaneous electrical stimulation at acupoints could reduce spasticity related to SCI. High-frequency electrical stimulation (100 Hz) produced an immediate antispastic effect, but required ongoing treatment, which was postulated to enhance the production of dynorphin in the anterior horn of the spinal cord that decreases the excitability of the motor neurons via kappa opiate receptor activation. These findings were confirmed by the study of Dong et al. [39] using 100 Hz stimulation to reduce spasticity in a rat acute SCI model.

## 10. Possible Mechanisms for Acupuncture's Effects

**Figure 1:**
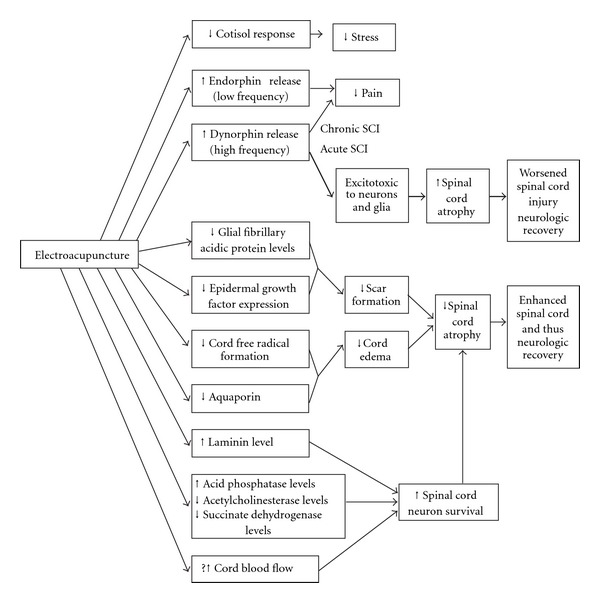
Possible mechanisms of acupuncture's effects in treating SCIs.

The potential mechanisms for acupuncture's beneficial clinical effects in treating the sequelae of SCI acutely are shown in Figure 1. In animal experimental models of acute SCI, electroacupuncture has been demonstrated to have a number of positive physiologic effects at the site of cord injury. Electroacupuncture produces reduced glial fibrillary acidic protein levels in the injured cord [40, 41], which serves to inhibit reactive astrocyte proliferation and reduce glial scar formation. Electroacupuncture also produces reduced epidermal growth factor receptor levels [41], also suggesting less scar formation. Electroacupuncture also reduces free radical formation [42], and down-regulates AQP-4 (aquaporin) expression after SCI [43] so as to inhibit spinal cord edema that can produce secondary spinal cord damage. Electoacupuncture also reduces spinal cord atrophy due to SCI with two-thirds reduction of anterior horn neuron loss [44] in acute SCI models in animals, and reduces the acute stress response of those animal as measured by the serum cortisol levels.

Enhanced spinal cord regeneration potential of electroacupuncture is implied by the findings of earlier and higher levels of laminin expression in the injured cord in animals treated with electroacupuncture [45], and by elevated levels of acid phosphatase during the recovery period [46, 47]. Wu et al. [47] reported that acupuncture reverses the elevations of acetylcholinesterase and succinate dehydrogenase and reductions of acid phosphatase seen in the anterior horn of the spinal cord involved by experimental SCI, which could inhibit or delay the deterioration (and possibly promote the recovery) of those anterior horn cell neurons.

Uchida and Hotta [48] have demonstrated acupuncture-like stimuli can increase cortical and uterine blood flow, and may provide another mechanism for acupuncture's effects in limiting damage and enhancing recovery of acute SCI. Electroacupuncture at low frequencies (*∼*2 Hz) produces endorphin release by the central nervous system [49] whose effect can be blocked by naloxone, while high frequency electroacupuncture causes release of dynorphins [10]. The effects of dynorphins on spinal cord neurons and glia are complex, as dynorphins may have neuroprotective or pro-apoptotic actions on spinal cord cells, depending on the distribution of kappa opioid receptors and the amount of dynorphin released [50]. Excessive dynorphin levels in acute SCI likely contribute to hyperalgesia as well as excitotoxic injury to neurons and glia, while in chronic SCI dynorphins released at normal physiologic concentrations through electroacupuncture may have analgesic and neuroprotective effects on spinal cord cells [50].

## 11. Conclusions

There is evidence that use of electroacupuncture in acute spinal cord injured subjects may significantly improve their long-term neurologic recovery including motor, sensory and bowel/bladder function. Acupuncture may even improve neurourologic function in spinal cord injured individuals with chronic neurogenic bladder, and may also be a useful adjunct in the management of their chronic neuropathic and musculoskeletal pain conditions. The acupuncture effects appear to result from stimulation of appropriate spinal cord segmental levels or peripheral nerves, and the known release of endogenous opioids at the spinal cord level produced by acupuncture treatment and electrical stimulation of peripheral nerves provides a plausible mechanism for its effects in pain relief and limiting SCI after acute trauma. At least theoretically, high frequency electroacupuncture (>100 Hz) in acute SCI may worsen excessive local dynorphin release that is excitotoxic to neurons and glial cells.

 The acupuncture literature on its use for treating the sequelae of acute and chronic SCI in humans is limited, and demonstrates wide variations in methodology both in terms of use of control interventions and types of acupuncture interventions. More recent studies, in general, are of higher methodologic quality and more closely adhere to the CONSORT criteria. Older studies, prior to 2000, tend to be more anecdotal, retrospective descriptions of the results of extensive clinical experience using acupuncture to treat the sequelae of human SCI. Though those retrospective studies do not provide the detailed patient or methodologic data that would permit rigorous scientific conclusions of acupuncture's effects in treating the sequelae of acute and chronic SCIs, they do provide important guidance on TCM concepts and point selections recommended by experts for treating the sequelae of SCI.

## Figures and Tables

**Table 1:** Prior studies of acupuncture's use in treating SCI sequelae.

Study	Number of patients	Randomized?	Acupuncture intervention	Control intervention	Standardized acupuncture?	Jadad score
All SCI problems						
Wong et al. [7]	100	Yes	75 Hz surface EA at SI-3 and BL-62 plus auricular acupoints plus usual care	Usual SCI rehabilitation care	Yes	2
Wang [11]	Not given	No	Wide variety	Not applicable	No	0, retrospective experience
Gao et al. [12]	261	No	Wide variety	Not applicable	No	0, retrospective experience
SCI bladder problems						
Cheng et al. [13]	60	Yes	EA 20–30 Hz at CV-3, CV-4, and bilateral BL-32 plus usual care	Usual care	Yes	2
Honjo et al. [14]	13	No	A manual stimulation at BL-33	Not applicable	Yes	1, no dropouts
Zhou et al. [15]	84	Yes	EA at Baliao and BL-35	EA at “acupoints routinely selected"	Yes	Insufficient information
Pain problems						
Nayak et al. [16]	20	No	A, no stimulation	Not applicable	No, some points standardized	1, efficacy study with 2 dropouts
Rapson [17]	36	No	EA	Not applicable	Yes	0, retrospective experience
Dyson-Hudson et al. [18]	17	Yes	A, manual stimulation	Sham superficial acupuncture	No, but selected from a group of points	5
Dyreflexia problems						
Averill et al. [19]	15	No	A, no stimulation	Not applicable	Yes, “specific points above and below the lesion"	1, “acupuncture analgesia study" no dropouts

Acupuncture (A) or electroacupuncture (EA).
